# Role of the malic enzyme in metabolism of the halotolerant methanotroph *Methylotuvimicrobium alcaliphilum* 20Z

**DOI:** 10.1371/journal.pone.0225054

**Published:** 2019-11-18

**Authors:** Olga N. Rozova, Ildar I. Mustakhimov, Sergei Y. But, Aleksandr S. Reshetnikov, Valentina N. Khmelenina

**Affiliations:** Federal Research Center “Pushchino Scientific Center for Biological Research of the Russian Academy of Sciences”, G.K. Skryabin Institute of Biochemistry and Physiology of Microorganisms, Russian Academy of Sciences, Pushchino, Moscow Region, Russia; University of Münster, GERMANY

## Abstract

The bacteria utilizing methane as a growth substrate (methanotrophs) are important constituents of the biosphere. Methanotrophs mitigate the emission of anthropogenic and natural greenhouse gas methane to the environment and are the promising agents for future biotechnologies. Many aspects of CH_4_ bioconversion by methanotrophs require further clarification. This study was aimed at characterizing the biochemical properties of the malic enzyme (Mae) from the halotolerant obligate methanotroph *Methylotuvimicrobium alcaliphilum* 20Z. The His_6_-tagged Mae was obtained by heterologous expression in *Escherichia coli* BL21 (DE3) and purified by affinity metal chelating chromatography. As determined by gel filtration and non-denaturating gradient gel electrophoresis, the molecular mass of the native enzyme is 260 kDa. The homotetrameric Mae (65x4 kDa) catalyzed an irreversible NAD^+^-dependent reaction of L-malate decarboxylation into pyruvate with a specific activity of 32 ± 2 units mg^-1^ and *K*_m_ value of 5.5 ± 0.8 mM for malate and 57 ± 5 μM for NAD^+^. The disruption of the *mae* gene by insertion mutagenesis resulted in a 20-fold increase in intracellular malate level in the mutant compared to the wild type strain. Based on both enzyme and mutant properties, we conclude that the malic enzyme is involved in the control of intracellular L-malate level in *Mtm*. *alcaliphilum* 20Z. Genomic analysis has revealed that Maes present in methanotrophs fall into two different clades in the amino acid-based phylogenetic tree, but no correlation of the division with taxonomic affiliations of the host bacteria was observed.

## Introduction

Aerobic bacteria utilizing methane as the sole energy and carbon source (methanotrophs) are widespread in the environment and play important roles in the global carbon and nitrogen cycles, including the control of emissions of anthropogenic and natural greenhouse gas methane [1]. They are promising organisms for future biotechnological applications as producers of various poly-carbon compounds from methane [2–6]. The applications of methanotrophs as biocatalysts require the deep understanding of their carbon and energy metabolism.

The currently known methanotrophs belong to the *Alpha-* and *Gamma-*classes of the phylum *Proteobacteria* and to the phylum *Verrucomicrobia*. Alphaproteobacterial (Type II) methanotrophs assimilate methane carbon as methylene tetrahydrofolate and CO_2_ through the serine pathway, sometimes in combination with the Calvin-Benson-Bassham (CBB) cycle [1,7], where C3 compounds are the first products. The members of the Gammaproteobacteria (Type I and Type X methanotrophs) assimilate carbon predominantly at the level of CH_2_O via the ribulose monophosphate (RuMP) cycle, where sugar phosphates are the first products, but also use the functional serine cycle [8,9]. Methanotrophs of the phylum Verrucomicrobia oxidize methane to CO_2_ and assimilate carbon via the CBB cycle [10,11].

Among the characterized methanotrophs, haloalkalitolerant species are especially promising biocatalysts due to their high growth rate and high resistance to contamination under the optimal culture conditions. *Methylotuvimicrobium alcaliphilum* 20Z, a Type I methanotroph, is a good candidate for methane bioconversion to valuable chemicals due to its ability to grow in a wide range of conditions (pH, salinity and methanol concentration) [12]. This strain is also an appropriate model system for deeper understanding of C1 metabolic pathways [5,13–16]. The whole genome of *Mtm*. *alcaliphilum* 20Z was also annotated and published [17].

**Fig 1:**
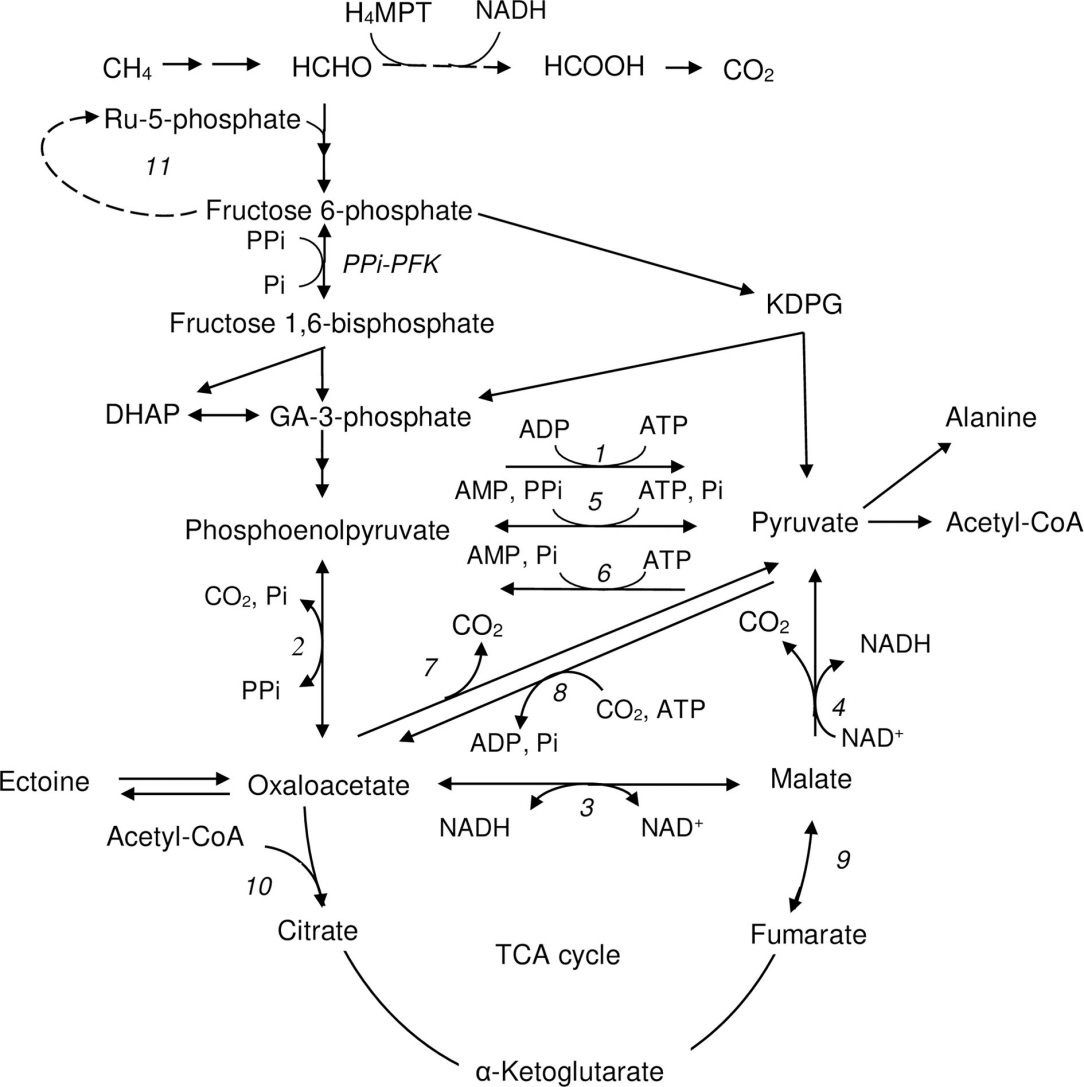
The central metabolism of *Mtm*. *alcaliphilum* 20Z. 1 –pyruvate kinase (accession number CCE24746), 2 –pyrophosphate type of phosphoenolpyruvate carboxykinase (WP_014148666), 3 –malate dehydrogenase (CCE24885), 4 –malic enzyme (CCE22813), 5 –pyruvate, phosphate dikinase (CCE23390), 6 –phosphoenolpyruvate synthase (CCE23522), 7 –oxaloacetate decarboxylase (CCE22241-β, CCE22242-α and CCE24338-γ), 8 –pyruvate carboxylase (CCE24021, CCE24020), 9 –fumarase (CCE21981; CCE23513), 10 –citrate synthase (CCE24690, CCE23049); 11 –reactions of the pentose phosphate pathway; PPi-PFK–PPi-dependent 6-phosphofructokinase (CCE21914); Ru-5phosphate–ribulose-5-phosphate, KDPG– 2-keto-3-deoxy-6-phosphogluconate, GA-3phosphate–glyceraldehyde-3-phosphate, DHAP–dihydroxyacetone phosphate.

In the RuMP pathway, the condensation of formaldehyde with ribulose 5-phosphate by hexulose phosphate synthase leads to the formation of hexulose 6-phosphate. In *Mtm*. *alcaliphilum* 20Z, there are at least three functional pathways for sugar phosphates cleavage: the modified pyrophosphate-dependent glycolysis, the Entner-Doudoroff and phosphoketolase pathways together forming C2-, C3-, and C4-compounds [18–20]. The key pathway node connecting the main pathways of C1 assimilation and central metabolism involves the interconversion of phosphoenolpyruvate (PEP), pyruvate, oxaloacetate (OAA) and malate. As judged from the genomic data, in *Mtm*. *alcaliphilum* 20Z these four compounds participate in at least 9 reactions (Fig 1). The strain 20Z encodes four enzymes interconverting PEP and pyruvate: pyruvate kinase A (PK II class, CCE24746, [18]), pyruvate kinase (CCE23546) of low identity (8%) with PK II, pyruvate phosphate dikinase (PPDK, CCE23390), and PEP synthase (CCE23522). PEP-carboxykinase (PEPCK, CCE23879) could reversibly carboxylate PEP into OAA [21,22]. The NAD^+^-malate dehydrogenase (MaDH, CCE24885) catalyzes the interconversion of OAA and malate [23]. There are two enzymes annotated in the genome of *Mtm*. *alcaliphilum* 20Z as candidates for interconversion of pyruvate and OAA: (i) two-subunit biotin-dependent pyruvate carboxylase (PC, CCE24021 and CCE24020) showing a 61–69% identity with the characterized enzyme from the methylotrophic bacterium *Methylobacillus flagellatus* [24] and (ii) hetero-subunit membrane-bond oxaloacetate decarboxylase (encoded by CCE22241-β, CCE22242-α and CCE24338-γ), which is highly identical with the oxaloacetate decarboxylases from *Vibrio cholera* [25]. Though the main function of the latter membrane-bound enzyme could be Na^+^-pumping [26, 27], it probably affects the intracellular pool of OAA.

Only malic enzyme (Mae, encoded by CCE22813) can perform the direct interconversion pyruvate and malate. In the case of reversibility of the reaction, this enzyme can be considered responsible for the replenishing of TCA intermediates [14, 16]. However, very few works are devoted to the role of Mae in methanotroph metabolism. Recently, based on the biochemical characterization of Mae from the alphaproteobacterial methanotroph *Methylosinus trichosporium* OB3b (Type II), we have proposed an essential role of this enzyme in the generation of NADPH required for biosynthetic processes [28].

Malic enzymes catalyze the oxidative decarboxylation of L‐malate to produce pyruvate and carbon dioxide coupled with NAD^+^ or NADP^+^ reduction. These enzymes require the presence of divalent cations (Mg^2+^ or Mn^2+^) for their activity. Malic enzymes are part of the family of structurally related proteins, which also includes malolactic enzymes and soluble oxaloacetate decarboxylases converting L-malate to L-lactate and OAA to pyruvate, respectively [29]. Three classes of Maes have been defined on the basis of their coenzyme specificity and ability to decarboxylate OAA: EC1.1.1.38 (NAD-dependent; decarboxylates added OAA), EC1.1.1.39 (NAD^+^-dependent; does not decarboxylate added OAA), and EC 1.1.1.40 (NADP^+^-dependent, some NADP^+^-Mae decarboxylate added OAA). Some NADP^+^-malic enzymes have an additional large C-domain highly identical to phosphoacetyltransferases. However, no activity reversibly transferring the acetyl group from acetyl phosphate to acetyl-CoA has been shown for any of the characterized chimeric malic enzymes. The additional C-domain promoted the correct folding of the enzyme and sometimes mediates inhibition by acetyl-CoA [28, 30, 31].

Maes have been found in representatives of all major biological divisions where they participate in diverse metabolic pathways such as photosynthesis, lipogenesis, and energy metabolism. The physiological functions of the enzyme can vary from organism to organism [32–34]. While many malic enzymes are able to catalyze the reversible reaction, their function of C3-carboxylation in bacteria remains doubtful due to the higher enzyme affinity towards malate than towards pyruvate [32, 35–36].

The gene presumably encoding Mae with low identity with the malic enzyme from *Ms*. *trichosporium* OB3b was found in the genome of *Mtm*. *alcaliphilum* 20Z [28]. In this study, we have obtained and characterized the recombinant Mae from the methanotroph. We have established that Mae from *Mtm*. *alcaliphilum* 20Z catalyzes the irreversible NAD^+^-dependent reaction of malate decarboxylation. We have shown that the strain lacking the malic enzyme accumulates an enhanced level of malate. The genomic analysis has revealed the presence of Mae-like genes in most methanotrophs sequenced to date. The orthologs of methanotrophic malic enzyme can be divided into two distinct clades in the phylogenetic tree, but this division is not consistent with the taxonomic affiliation of the host bacteria.

## Materials and methods

### Bacteria and growth conditions

*Methylotuvimicrobium alcaliphilum* 20Z (rename *Methylomicrobium*) (VKMB-2133, NCIMB14124) was cultivated at 30°C in nitrate mineral medium 2P with an addition of 3% NaCl and 0.1 M sodium carbonate buffer (pH 9.0) under methane-air atmosphere (1:1) or in the presence of methanol (0.3%) [37]. *Escherichia coli* strains BL21 (DE3), Top 10 and S17-1 (Novagen) were grown at 37°C on selective Luria–Bertani (LB) agar or in LB broth with constant shaking (150 rpm). For cultivation of the plasmid-bearing *E*. *coli*, 100 μg mL^-1^ ampicillin, kanamycin (50 μg mL^-1^), tetracycline (50 μg mL^-1^) or gentamicin (4 μg mL^-1^) were added to the medium if necessary.

### DNA manipulations

Plasmid isolation, digestion by restriction enzymes, agarose gel electrophoresis, ligation and transformation of *E*. *coli* cells were performed according to Sambrook and Russell [38]. Restriction enzymes, T4 DNA-ligase, *Pfu*, *Taq* and T4 DNA polymerases, dNTPs and Page Ruler Prestained Protein Ladder for SDS-PAGE were purchased from Thermo Scientific.

### Cloning and purification of the malic enzyme

The *mae* gene of 1689 bp (CCE22813) from the genomic DNA of *Mtm*. *alcaliphilum* 20Z was amplified by PCR using the primers based on the sequence from GenBank (accession number FO082060): forward Mae-Acc-Nde-F (5'-AAGGTACCAGGAGATTCCATATGACAGCAATGTCGAAAACTCCTCT) and reverse Mae-Xho-R (5'-ATCTCGAGAAAACAAACCCGCTTATAGTGCCGAT), and containing recognition sites for endonucleases *Acc65I*, *Nde*I and *Xho*I, respectively. The PCR product was cloned into the pZeRO vector (Invitrogen). The correct insertion was verified by sequencing. The gene was cut out from pZeRO:mae with *Nde*I and *Xho*I and ligated into pET22b(+) plasmid. The resultant vector pET22b:mae was transferred into *E*. *coli* BL21 (DE3) cells. The transformed cells of *E*. *coli* were grown at 37°C in a liquid LB medium containing 100 μg mL^-1^ ampicillin; enzyme expression was induced by the addition of 0.5 mM isopropyl-1-thio-β-D-galactopyranoside (IPTG) at OD_600_ = 0.6–0.8. After 15-h growth at 18°C, the cells were harvested by centrifugation (30 min at 8°C and 5000 g) and stored at -20°C. The His_6_-tagged protein was purified by affinity chromatography on a Ni^2+^-nitrilotriacetic acid (Ni-NTA) column as described earlier [39]. The purified enzyme was stored in 40% glycerol at -20°C.

### Mutant generation

The strain deficient in the *mae* gene was obtained by insertion of the kanamycin resistance gene. Briefly, the *mae* sequence was cut out from pZeRO:mae with endonucleases *Xho*I (blunting with T4 DNA polymerase) and *Acc65I* (S1 Fig). The obtained 1692 bp fragment was ligated into a suicidal vector pCM184 cut with *SacI* (blunted with T4 DNA polymerase) and *Acc65I*. Then the kanamycin resistance gene (1200 bp) cut out of the pCM184 vector with endonucleases *PstI* and blunted with T4 DNA polymerase was inserted into the middle part of the *mae* sequence between the blunting sites for *AsuII* and *SacII* endonucleases instead of 591 bp. The resultant plasmid pCM:*mae-Km* was transferred into *E*. *coli* S17-1 and conjugated into *Mm*. *alcaliphilum* 20Z. Mutants were selected on an agar medium containing 0.3% methanol and 100 μg mL^-1^ of kanamycin. Double-crossover *mae*^*-*^ mutants were identified by the product of 2330 bp (vs. wild type 1690 bp) using the diagnostic PCR test and the Mae-Acc-Nde-F and Mae-Xho-R primers (S2 Fig).

### Complementation of the *mae*^*-*^ mutation

The *mae* gene with the Shine–Dalgarno sequence AGGAGATTCCAT was cut out of pZeRO:mae with endonucleases *Acc65*I and *Xho*I. The ~1.7-kb fragment was cloned into the pMHA200pmxa vector resulting in the pMHApmxa:mae vector to provide the expression of *mae* under the control of methanol dehydrogenase promoter P*mxa* [13]. *E*. *coli* S17-1 was used for transconjugation of pMHA200pmxa:mae into the *mae*^*-*^ mutant as described for mutant generation. The *Mtm*. *alcaliphilum mae*^*-*^ mutant with the pMHApmxa:mae plasmid (denoted as *mae*^-^::*mae*) was selected on a solid medium containing 0.3% methanol, 100 μg mL^-1^ kanamycin and 10 μg mL^-1^ gentamycin and the inserted plasmid was verified by the diagnostic PCR with the Mae-Acc-Nde-F and Mae-Xho-R primers (S2 Fig).

### Determination of molecular masses of Mae

The molecular mass of the recombinant Mae was estimated using non-denaturating gel electrophoresis in the pore-limiting gradient of polyacrylamide (4–30%) and gel filtration chromatography with a XK 16/100 Superdex 200 column (GE Healthcare) equilibrated with 0.02 M Tris-HCl (pH 7.0) containing 0.5 M NaCl. The flow rate was 1 mL min^-1^, and the proteins were detected by monitoring at 280 nm. The protein standards were carbonic anhydrase (29 kDa), albumin (66 kDa), alcohol dehydrogenase (150 kDa), β-amylase (200 kDa), and apoferritin (443 kDa).

### Enzyme assays

The activity of Mae was assayed by measuring NAD^+^ reduction at 340 nm in the reaction mixture (1 mL) containing: 50 mM Tris-HCl buffer, pH 9.0; 2.5 mM MgCl_2_; 0.3 mM NAD^+^, 10 mM malic acid disodium salt, and 4 μg of the recombinant enzyme. The ability of *Mtm*. *alcaliphilum* Mae to use NADP^+^ as a cofactor was tested using NADP^+^ instead of NAD^+^. The product of the Mae reaction was tested with alanine dehydrogenase from *Bacillus subtilis* using pyruvate but not lactate for activity [40]. After the reaction was stopped, 5 mM NH_4_Cl and 10 U of alanine dehydrogenase were added and NADH oxidation was observed. The pyruvate carboxylation ability was tested in 1 mL of the reaction mixture containing 50 mM buffer (pH 6.0 to 10.0), 2.5 mM MgCl_2_, 0.25 mM NAD(P)H, 5–50 mM sodium pyruvate, and ~10–50 μg Mae. 50 mM KHCO_3_, NaHCO_3_ or Na_2_CO_3_ was tested as a CO_2_ source.

The oxaloacetate decarboxylation activity by *Mtm*. *alcaliphilum* Mae was tested by measuring the decrease in absorbance at 280 nm [41] in the reaction mixture containing 50 mM MES-NaOH buffer (pH 5.0) or Tris–HCl (pH 8.0) buffer, 1–10 mM oxaloacetate, 2.5 mM MgCl_2_, and 50 μg of Mae in the presence or absence of NAD^+^ (0.25 mM). Also, the formation of pyruvate in this reaction was tested by HPLC on a ReprosilPur c18AQ column (5 μm, 250 × 10 mm) (Dr. Maisch, Germany) using 1 mM H_2_SO_4_ and 8 mM Na_2_SO_4_ as the mobile phase at 25°C and a flow rate of 1 mL min^-1^.

Glycine-NaOH (pH 9.0–10.5), CHES–NaOH (pH 8.5–10.0), Tris–HCl (pH 7.6–8.9), K-phosphate (pH 6.0–8.0) and MES-NaOH (pH 5.0–7.0) at a concentration of 50 mM were used to study pH dependence of the enzyme activity. Potential effectors of Mae were tested: fructose, glucose, glucose 6-phosphate, fructose 6-phosphate, fructose 1,6-bisphosphate at 5 mM concentration; sodium pyruvate, phosphoenolpyruvate, oxaloacetate, fumarate, α-ketoglutarate, isocitrate, citrate, succinate, hydroxypyruvate; glutamate, aspartate, serine (1 mM); ATP, ADP, AMP, PPi, acetyl phosphate (2 mM), CoA (0.1 mM), and acetyl-CoA (0.2 mM). All compounds were obtained from AppliChem, Sigma-Aldrich, or Santa Cruz Biotechnology.

To test the effect of monovalent and divalent cations on the enzyme activity, the aqueous stock solutions of KCl, NH_4_Cl, NaCl (50 mM final concentration), MgCl_2_, MnCl_2_, CuCl_2_, RbCl_2_, CdCl_2_, NiCl_2_, SnCl_3_, CoCl_2_, BaCl_2_, ZnCl_2_ or CaCl_2_ (1 mM) were added in to reaction mixture. To check the thermal stability, the aliquots of the enzyme were incubated at 30, 40, 50, 60 and 70°C from 5 min to 3 h, then the residual activity was determined at 30°C. To search for optimal temperature, the reaction was carried out at 10–70°C. Apparent *K*_m_ and *V*_max_ values were calculated using SigmaPlot (version 10). Protein concentrations were assayed by the modified Lowry method [42]. The NADH oxidation/formation rates were recorded at 340 nm with a UV-1700 spectrophotometer (Shimadzu, Japan).

### Extraction and analysis of metabolites

Exponentially grown cells (about 250 mg) were suspended in 1 ml of 80% methanol and disrupted by sonication for 1 min in 20-s bursts with 30-s cooling on ice between the bursts with a MSE sonicator (England). The suspension was centrifuged for 5 min at 10,000 g. The supernatant was dried under vacuum with Concentrator 5301 (Eppendorf, Germany). The dried extract was re-dissolved in 100 μl of deionized H_2_O. The solution was purified with cool chloroform and the organic acids in the water fraction were analyzed by HPLC (Shimadzu, Japan). Organic compounds were separated on a Repro-Gel H+ column (9 μm, 250x8 mm) (Dr. Maisch, Germany) using 1 mM H_2_SO_4_, 0.5 mL min^-1^ at 50°C for elution and on a Reprosil-Pur c18-AQ column as described above. The peaks of compounds were registered at 210 nm.

### Enzyme assays in the cell-free extract

About 250 mg of exponentially grown cells were suspended in 1 mL 0.05 M Tris-HCl buffer, pH 8.0, and disrupted by sonication for 2 min in 20 s bursts with 30 s cooling on ice between the bursts by MSE sonicator (England). The suspension was centrifuged for 10 min at 14,500 g. The malic enzyme and malate dehydrogenase activities were tested in the following reaction mixture (1 mL): 50 mM Tris-HCl (pH 9.0), 0.5 mM NAD^+^, 10 mM malate, 50 μL cell-free extract (~3 mg of protein) from the wild type strain, the *mae*^-^ strain, or the strain with complementation of the *mae*^*-*^ mutation. The reaction was carried out for 30 min at 30°C and then stopped by cooling to 4°C. The reaction products were analyzed by HPLC (Shimadzu, Japan) with a Repro-Gel H+ column (9 μm, 250 x 8 mm) as described above. The reaction mixtures without the cell-free extract or the substrate were used as controls. The reverse malate dehydrogenase activity was tested in the following reaction mixture: 50 mM Tris-HCl (pH 9.0), 0.3 mM NADH, 5 mM oxaloacetate, 50 μL cell-free extract of the wild type strain, the *mae*^*-*^ strain, or the strain with complementation of the *mae*^*-*^ mutation. The fumarase activity was tested in the reaction mixture containing 50 mM Tris-HCl (pH 8.0), 2.5 mM fumarate, 50 μL cell-free extracts of the wild type strain, or the *mae*^-^ strain, or the strain with complementation of the *mae*^*-*^ mutation. The control reaction mixtures did not contain fumarate or cell-free extract.

### Sequence analysis

The sequences from the NCBI database (http://www.ncbi.nlm.nih.gov) were obtained by BLAST searches. The alignments of amino acid sequences of different malic enzymes and the phylogenetic analysis were performed using MEGA 6 and the Neighbor-Joining model [43]. Minor corrections in the alignments were done manually. The branches corresponding to partitions reproduced in less than 50% bootstrap replicates were not indicated. There were 588 informative positions in the final dataset.

## Results

### Purification of Mae from *Mtm*. *alcaliphilum* 20Z

**Fig 2:**
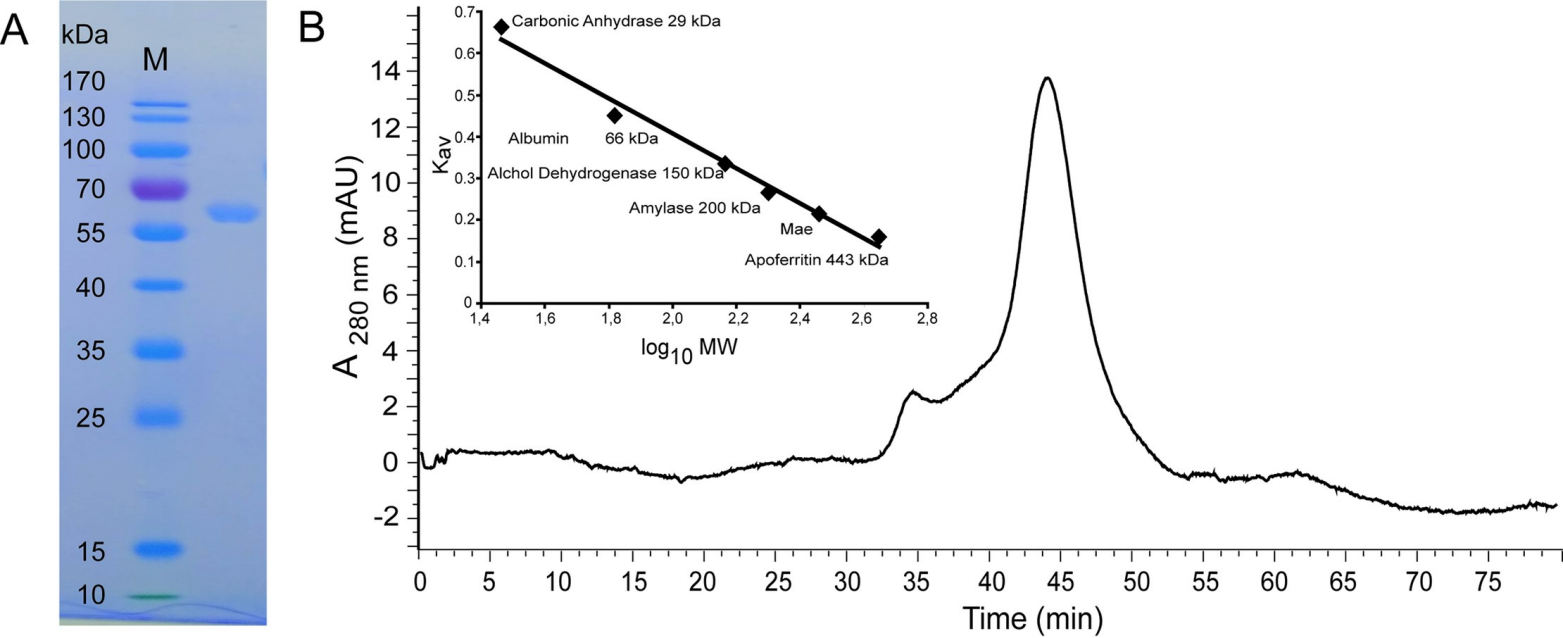
Purification and oligomeric state determination of the recombinant *Mtm*. *alcaliphilum* Mae. (A) The protein purity was determined by 12% SDS-PAGE. M, markers of molecular mass. (B) Molecular mass determination by gel filtration chromatography. The proteins were detected by monitoring their absorbance at 280 nm. The molecular mass standard curve with Mae is inseted.

*Mtm*. *alcaliphilum mae* was cloned in the pET22b(+) vector and successfully expressed in the cells of *E*. *coli* BL21 (DE3). The His_6_-tagged protein was purified by Ni^2+^-bounded affinity chromatography. SDS-PAGE under denaturing conditions showed the homogeneity of the enzyme; its apparent molecular mass ~65 kDa (Fig 2A) was close to the value predicted from the coding sequence (63.4 kDa). According to the native gradient electrophoresis (S3 Fig), the estimated molecular mass of the enzyme was 260 kDa, suggesting its homotetrameric structure. A single symmetric peak with a molecular mass of approximately 260 kDa was observed in the gel filtration experiment thus confirming that Mae is a homotetramer in solution (Fig 2B).

### The kinetic properties of Mae-His_6_

The recombinant Mae from *Mtm*. *alcaliphilum* 20Z catalyzed NAD^+^ reduction in the presence of malate and Mg^2+^. Since the methanotrophic enzyme displayed an appreciable sequence similarity (40% identity) with a malolactic enzyme from lactobacteria [44], the product of the Mae reaction was tested using alanine dehydrogenase from *B*. *subtilis* and was found to be pyruvate. The reaction was strongly dependent on NAD^+^, whereas NADP^+^ was not a cofactor. The enzyme did not catalyze the reaction of pyruvate carboxylation under any conditions tested as described in Materials and Methods: in the pH range from pH 6.0 to pH 10.0, at a pyruvate concentration of 5–50 mM, a protein content of 10–50 μg, and at different bicarbonate concentrations (up to 50 mM). It did not catalyze oxaloacetate decarboxylation since no decrease in absorbance at 280 nm and pyruvate formation were found.

Mae was active in the pH range from pH 6.0 to pH 10.5, displaying the maximum activity at pH 9.0 (S4 Fig). The temperature optimum of the Mae reaction was 55 ^о^C (S5 Fig). However, incubation of the protein within a temperature range from 30 to 60°C led to progressive inactivation of the enzyme. Residual activity was 80% after 3-hour exposure at 30°C; 50% of the activity was lost after 30-min heating at 40°C; the enzyme was fully inactivated after 20-min incubation at 50°C or after 5-min incubation at 60°C.

All kinetic parameters of the *Mtm*. *alcaliphilum* Mae were determined at pH 9.0, which is optimal pH for the enzyme, and at 30°C which is optimal growth temperature for the bacterium. The dependence of the Mae activity on substrate concentration obeyed the Michaelis–Menten kinetics. Under these conditions, the activity of the *Mtm*. *alcaliphilum* Mae was found to be 32 ± 2 U per mg of protein. The enzyme had a low affinity to malate (the apparent Km = 5.5 ± 0.8 mM) but a very high affinity to NAD^+^ (the apparent Km = 57 ± 5 μM) (Table 1).

**Table 1:** Some properties of Mae from methanotrophs.

Properties	*Mtm*. *alcaliphilum* 20Z [this work]	*Ms*. *trichosporium* OB3b [27]
**Subunit molecular mass, kDa**	260 (65 x 4)	480 (80×6)
**pH-optimum**	9.0	7.5
***T***_**opt**_**,°C**	50	65
Inhibitors*	50 mM NH_4_^+^ (56%), Zn^2+^ (100%), Cd^2+^ (100%)	1 mM Hydroxypyruvate (55%) 0.2 mM Acetyl-CoA (24%)
***V***_**max**_**, U mg**^**-1**^ **(malate → pyruvate)**	32 ± 2	36
**Vmax, U mg**^**-1**^ **(pyruvate → malate)**	-**	8
***K***_**m**_:		
**Malate, mM**	5.5 ± 0.8	2.7
**NAD**^**+**^, μ**M**	57 ± 5	-
**NADP**^**+**^, μ**M**	-	64
**Pyruvate, mM**	-	6
**NADPH, μM**	-	47

* Residual activity (%) of Mae in the presence of the inhibitors

** The activity was not found.

### Effects of different metabolites and metals

The activity of Mae from *Mtm*. *alcaliphilum* 20Z was strongly dependent on Mn^2+^ or Mg^2+^, which is a common feature of all known malic enzymes. K^+^ cations at a concentration of 50 mM had no appreciable effect, whereas Na^+^ and NH_4_^+^ inhibited the enzyme activity by 30 and 40%, respectively (Table 2). In the presence of 1 mM Mn^2+^, Sn^3+^ at the same concentration reduced the activity by 40%, whereas Zn^2+^ or Cd^2+^ completely inhibited the enzyme (S1 Table).

**Table 2:** Influence of mono- and divalent ions on the activity of Mae from *Mtm*. *alcaliphilum* 20Z. The monovalent ions at a final concentration of 50 mM and divalent ions at a final concentration of 1 mM were used for testing.

Ions	Residual activity, %
Without ions	< 0,01
K^+^	< 0,01
Mg^2+^	100 ± 2
Mn^2+^	132 ± 2
Co^2+^	44 ± 1
K^+^, Mg^2+^	103 ± 2
Na^+^, Mg^2+^	71 ± 5
NH_4_^+^, Mg^2+^	56 ± 3

The analysis did not reveal significant effect of organic metabolites on the Mae activity (Table 3). ATP and PPi (2 mM) moderately inhibited the enzyme activity. However, these inhibitory effects were completely abolished upon increase of Mg^2+^ concentration, suggesting the chelating effects of these phosphates.

**Table 3:** The activity of *Mtm*. *alcaliphilum* Mae in the presence of some metabolites (residual activity, %).

Potential effectors (Concentration)	Activity
No effectors	100
Oxaloacetate (1 mM)	92 ± 3
Isocitrate (1 mM)	96 ± 1
Citrate (1 mM)	99 ± 1
α-Ketoglutarate (1 mM)	105 ± 2
Succinate (1 mM)	110 ± 2
Fumarate (1 mM)	109 ± 4
Glutamate (1 mM)	108 ± 3
Posphoenolpyruvate (1 mM)	96 ± 4
Pyruvate (1 mM)	100 ± 2
Hydroxypyruvate (1 mM)	78 ± 3
Serine (1 mM)	107 ± 2
Aspartate (1 mM)	102 ± 4
Glucose (5 mM)	87 ± 3
Glucose-6-phosphate (5 mM)	111 ± 2
Fructose (5 mM)	92 ± 3
Fructose-6-phosphate (5 mM)	104 ± 1
Fructose-1,6-phosphate (5 mM)	88 ± 4
ATP (2 mM)	83 ± 3
ATP (2 mМ), MgCl_2_ (5 mМ)	98 ± 2
ADP (2 mM)	87 ± 1
AMP (2 mM)	88 ± 4
PPi (2 mM)	55 ± 5
PPi (2 mМ), MgCl_2_ (5 mМ)	115 ± 3
CoA (0.1 mM)	90 ± 4
Acetyl-CoA (0.2 mM)	86 ± 3
Acetyl phosphate (2 mM)	94 ± 1

### Phenotypic characteristics of the *mae*^*-*^ mutant

**Fig 3:**
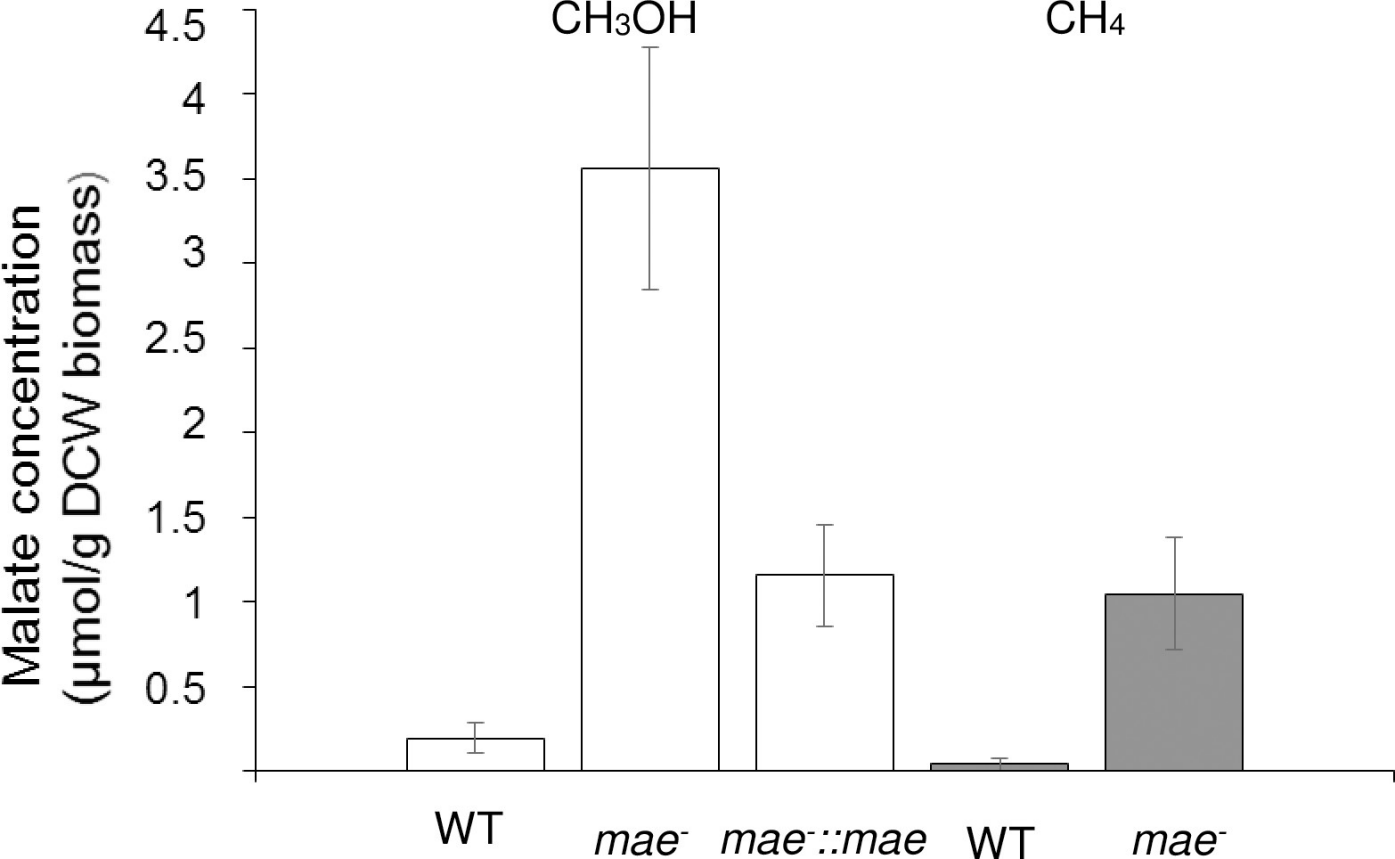
Intracellular concentration of malate in *Mtm*. *alcaliphilum* 20Z grown on methanol (white column) and under methane (grey column). WT, the wild type strain; *mae*^*-*^, the mutant strain; *mae*^*-*^::*mae*, the strain with complementation of the *mae*^*-*^ mutation.

Disruption of the gene encoding the malic enzyme did not affect the growth rate under methane or in methanol as carbon substrate at medium salinity 1, 3 or 6% NaCl. However, in the cells of the mutant strain grown under methane or on methanol, malate concentration was 1.0 ± 0.3 μmol per g of dry cell weight (DCW) or up to 4.0 ± 0.7 μmol per g DCW, respectively (Fig 3). These values were ~20-fold higher than in the initial culture, since the cells of the wild type strain grown under methane contained 0.040 ± 0.009 μmol of malate per g DCW and the methanol-grown culture accumulated 0.20 ± 0.09 μmol of malate per g DCW. These data suggest that in the strain 20Z the malate outflow from the TCA cycle can proceed via the Mae reaction.

The plasmid pMHA200pmxa:mae carrying *mae* under the constitutive methanol dehydrogenase promoter pmxa was constructed and introduced into the *mae*^*-*^ mutant strain by trans-conjugation. The transformed strain (*mae*^*-*^::*mae*) grown on methanol accumulated four times less malate (~1.0 ± 0.3 μmol per g DCW) compared to the mutant strain (Fig 3).

### Analysis of metabolites formed from C4-dicarboxylic acids by cell-free extracts

**Fig 4:**
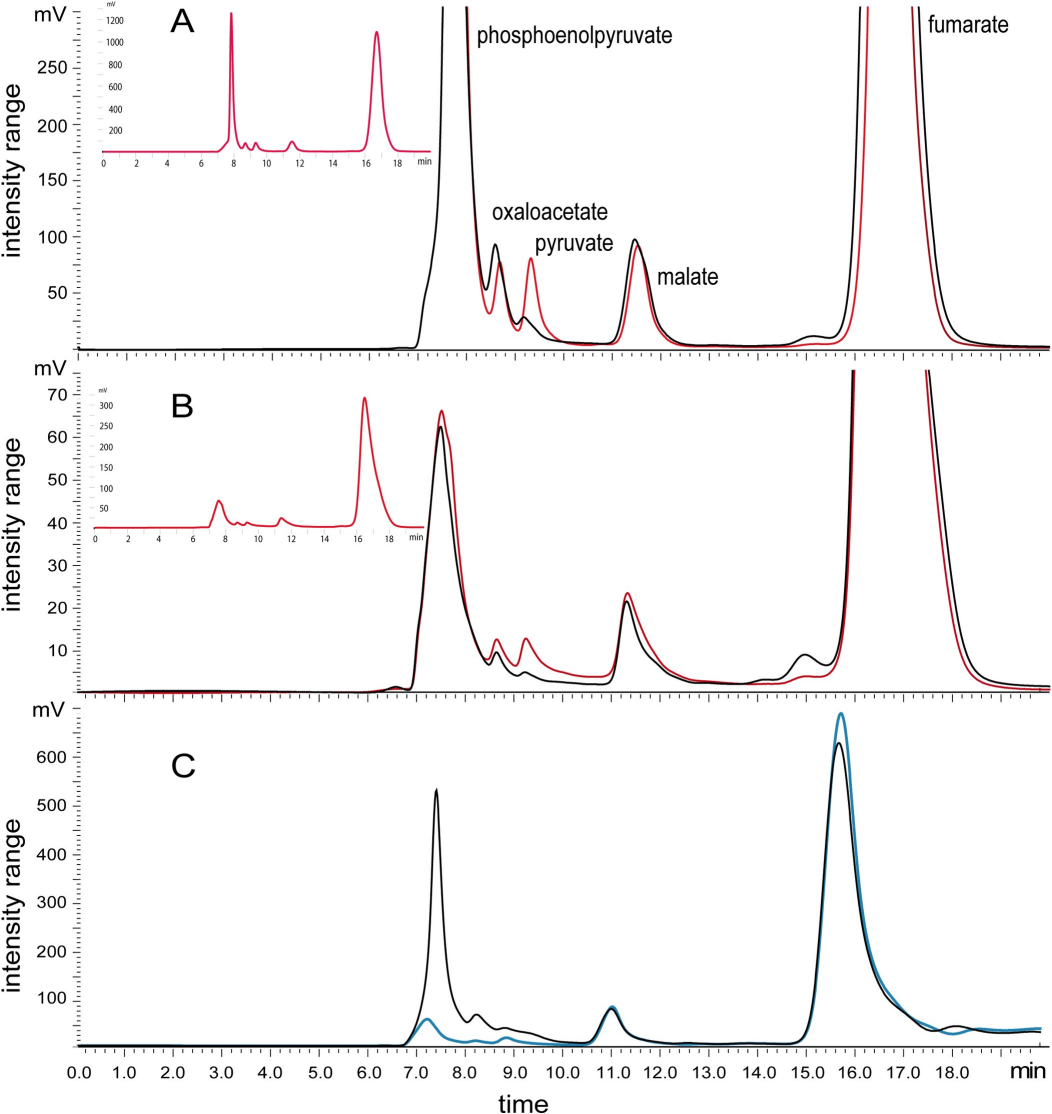
Analysis of products of the reactions catalyzed by cell-free extracts of the WT strain and the *mae*^-^ mutant. (A) Malate and NAD^+^ were substrates for the reactions. (B) Fumarate was a substrate for the reactions. The red line represents reaction products in cell-free extract of the WT strain and the black line represents reaction products in cell-free extract of the *mae*^-^ mutant. (C) The products of reactions of *mae*^-^ mutant extracts in the presence of malate (blue line) or malate + NAD^+^ (black line). The inserts are scaled-down chromatograms with full-size peaks of the tested compounds. The reaction products were analyzed by HPLC with a Repro-Gel H^+^ column using 1 mM H_2_SO_4_, 0.5 mL min^-1^ at 50°C for elution and registered at 210 nm.

The cell-free extracts from *Mtm*. *alcaliphilum* 20Z and its *mae*^*-*^ mutant were incubated for 30 min in the presence of malate or fumarate; the products of transformation of C4-dicarboxylic acids were analyzed by HPLC. The extract of the wild-type strain catalyzed the production of fumarate, OAA, PEP and pyruvate from malate in the presence of NAD^+^ (Fig 4): the conversion of malate into fumarate, the oxidation of malate to OAA, the decarboxylation of OAA to PEP, and the decarboxylation of malate to pyruvate. In contrast, only traces of pyruvate were found among the products of malate conversion by the extracts obtained from the *mae*^-^ strain (Fig 4A and 4B). Notably, pyruvate accumulated in the extract of the *mae*^*-*^::*mae* strain (S6B Fig). The accumulation of PEP was detected in the incubation mixture only if NAD^+^ (0.5 mM) was added as a co-substrate (Figs 4C and S6C). These data are in accordance with malate conversion to PEP via the sequential reactions catalyzed by MaDH and PPi-PEPCK and pyruvate formation by Mae. This reaction required the presence of NAD^+^ due to the low affinity of MaDH for NAD^+^ (*K*_m_ = 450 μM) [23]. Nevertheless, the low NAD^+^ concentration present in the cell-free extracts was still sufficient to provide the Mae activity (*K*_m_ to NAD^+^ = 57 μM) (Fig 4B). However, in the cell-free extract incubated with 5 mM OAA, the expected transformation products were not detected, probably because of the inhibitory effect of OAA (S6D Fig).

### Genomic analysis of the malic enzymes in methanotrophs

**Fig 5:**
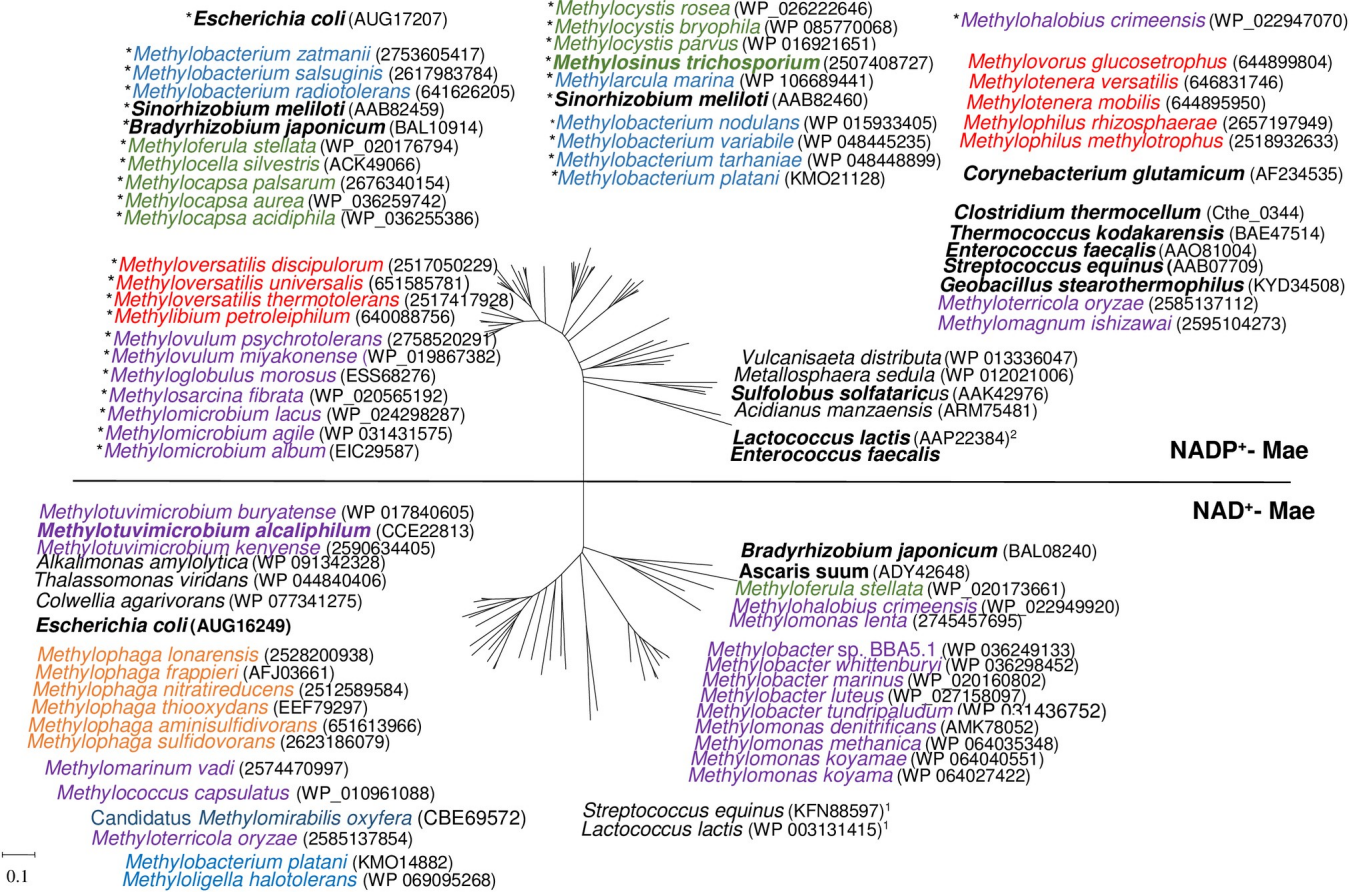
Phylogenetic tree of the malic enzymes from methylotrophic bacteria. The tree was constructed using the Neighbor-Joining algorithm as part of the MEGA 6 package (computed from 100 independent trials). Microorganisms possessing chimeric NADP^+^-dependent malic enzymes are marked with asterisks. Methylotrophs differently colored are representatives of: gammaproteobacterial methanotrophs (lilac), gammaproteobacterial non-methanotrophic methylotrophs (orange), alphaproteobacterial methanotrophs (green), alphaproteobacterial non-methanotrophic methylotrophs (blue), betaproteobacterial methylotrophs (red), anaerobic methanotrophs from NC10 phylum (dark-blue). Non-methylotrophic bacteria are black. The microorganisms with the characterized enzyme are in bold [28–31, 45–53]. The protein sequence accession numbers from the GeneBank or https://img.jgi.doe.gov are given in brackets. The scale bar corresponds to the number of substitutions per site. Only *mae*-fragments of the chimeric proteins (without part-fragment) were used for analysis. ^1^ –malolactic enzyme; ^2^ –oxaloacetate decarboxylase.

Mae from *Mtm*. *alcaliphilum* 20Z and the enzyme from *Ms*. *trichosporium* OB3b have a 10% identity of translated amino acid sequences and fall into different clades of the malic enzymes (Fig 5). Phylogenetic analysis showed that the *Mtm*. *alcaliphilum* Mae is clustered together with the malic enzymes from most other gammaproteobacterial methanotrophs (Fig 5). This cluster also includes the characterized NAD^+^-Mae from *E*. *coli* and *Bradyrhizobium japonicum* [30,45], as well as the malolactic enzyme from *Streptococcus equinus* and *Lactococcus lactis*. The NADP^+^-Mae from *Ms*. *trichosporium* OB3b and from all other alphaproteobacterial methanotrophs are clustered together on the phylogenetic tree (Fig 5). Some other gammaproteobacterial methanotrophs (of the genera *Methyloglobus*, *Methylohalobius*, *Methylovulum*, *Methylosarcina* and *Methylomicrobium*) possess the genes encoding NADP^+^-Mae. Two species, *Methyloterricola oryzae* and *Mh*. *cremeensis*, have both NAD^+^- and NADP^+^-Mae encoding genes (Fig 5), whereas several methanotrophs (*Methylogaea oryzae*, *Methylocaldum szegediense* O-12 and *Methylacidiphilum infernorum*) do not encode malic enzyme at all.

Like all Maes from both clades, the enzyme from *Mtm*. *alcaliphilum* has two highly conserved pyridine nucleotide-binding domains GXGXXG/A (Rossmann fold [54,55]) (149-VVTDGERVLGLGDQG-163 and 300-GAGSAG305) (S7 Fig). The presence of these conserved residues suggests that the malic enzymes implement the same catalytic mechanism including two steps: the dehydrogenation of malate to produce oxaloacetate and the decarboxylation of oxaloacetate to produce pyruvate. However, not all Mae are able to decarboxylate oxaloacetate.

## Discussion

In this study we have characterized for the first time the malic enzyme from a Type I methanotrophic bacterium. Mae from *Mtm*. *alcaliphilum* 20Z is specific to NAD^+^ and unable to decarboxylate OAA; therefore, it is a representative of the EC 1.1.1.39 class of the malic enzymes. The *Mtm*. *alcaliphilum* Mae shows low identity (<10%) with the earlier characterized NADP^+^-Mae from the Type II methanotroph *Ms*. *trichosporium* OB3b [28]. In addition to different cofactor specificity, Maes from the two methanotrophs have different catalytic properties, protein structure and metabolic functions (Table 1). The activities of both enzymes are strictly dependent on Mn^2+^ or Mg^2+^; however, the activity of Mae from *Ms*. *trichosporium* substantially increases in the co-presence of Mg^2+^ and K^+^ (or NH_4_^+^), whereas the Mae from *Mtm*. *alcaliphilum* is inhibited by NH_4_^+^ but not significantly influenced by K^+^ cations (Tables 1 and 2). The Mae from *Mtm*. *alcaliphilum* 20Z is a homotetramer consisting of 65-kDa subunits, whereas the *Ms*. *trichosporium* NADP^+^-Mae is a large homohexamer with a 80-kDa subunit possessesing a large carboxyl-terminal region homologous to phosphotransacetylase (EC 2.3.1.8). The chimeric NAD(P)^+^- or NADP^+^-Mae can also be identified by sequence homology in many gram-negative bacteria [31, 48].

Different cofactor specificity of Maes correlates with the central metabolic pathways of the two methanotrophs. In *Ms*. *trichosporium* OB3b, the NADP^+^-Mae supplies NADPH essential for the synthesis of steroids and fatty acids. Such “lipogenic” function was earlier proposed for NADP^+^-Mae from other bacteria [30–32, 56]. This is relevant for the Type II methanotroph lacking the oxidative pentose phosphate pathway as a NADPH provider, whereas the Type I methanotroph *Mtm*. *alcaliphilum* 20Z possesses the active enzymes of the pentose phosphate pathway. The alternative routes for NADPH generation via NADP^+^-dependent isocitrate dehydrogenases and the tetrahydromethanopterin pathway are nevertheless present in both bacteria [8, 57].

The most of malic enzymes characterized to date are positively and/or negatively regulated by a variety of organic compounds of the central metabolism. As is shown, hydroxypyruvate and acetyl-CoA inhibited the NADP^+^-Mae from *Ms*. *trichosporium* OB3b [28]. CoA, acetyl-P, palmitoyl-CoA and OAA inhibited but aspartate activated the NAD^+^-Mae from *E*. *coli* [30]. The TCA cycle intermediates had an inhibitory effect but glutamate and metabolites of glycolysis activated the NAD^+^-Mae from *Bradyrhizobium japonicum* [45]. In contrast, only ATP and PPi (2 mM) exhibited a moderate inhibitory effect on the *Mtm*. *alcaliphilum* NAD^+^-Mae. However, the inhibition may be due to the metal chelating effect of the phosphates, since the inhibition was completely abolished in response to the increase in magnesium concentration.

The negligible effect of organic compounds on the enzyme activity and very high *K*_m_ for malate imply that the Mae reaction in *Mtm*. *alcaliphilum* 20Z could be a mechanism for removing of malate excess. Inhibition of the Mae activity by NH_4_^+^ corroborates the catabolic function of the enzyme. The accumulation of malate might be a result of its synthesis through the oxidative TCA cycle (from fumarate) and the reduction of OAA entering from C3-carboxylation. The genome of *Mtm*. *alcaliphilum* 20Z encodes two isoforms of fumarase (CCE21981 and CCE23513) catalyzing the reversible transformation of fumarate into malate. At least two C3-carboxylation enzymes: pyruvate carboxylase (EC 6.4.1.1) and pyrophosphate-dependent PEP-carboxykinase (EC 4.1.1.38), are involved in anaplerosis to form OAA. The replenishment of the OAA pool is necessary to start the oxidative part of the TCA cycle and glutamate synthesis and because of the OAA outflow for the biosynthesis of aspartate, which is an ectoine precursor. In the *Mtm*. *alcaliphilum* 20Z, the high affinity of MaDH for NADH (Km 25 μM), in contrast to its low affinity for NAD^+^ (Km 450 μM) [23], along the high affinity of Mae for NAD^+^ (Km 57 μM) suggest that the reduction of OAA to malate and the subsequent oxidative decarboxylation of malate to pyruvate are favorable processes regulated by the intracellular NAD^+^/NADH balance. In turn, the outflow of C4-dicarboxylic acids from the TCA cycle becomes necessary at least in the case of ectoine degradation, which can occasionally result from external salinity fluctuation [58]. The removal of C4-dicarbonic acid from the TCA cycle in the methanotroph resembles the process known as cataplerosis occurring in plants or in bacteria growing in the presence of amino acids, when the TCA cycle cannot fully oxidize their carbon skeletons [59].

Interestingly, the methanol-grown cells accumulated almost fourfold more malate compared to the cells growing under methane. The difference in malate accumulation corroborates different roles of the TCA cycle in the methane- and methanol-growing cultures as has been demonstrated earlier for *Methylotuvimicrobium* [14, 60,61]. In *Mtm*. *buryatense* 5GB1 the flux distribution shift in methanol growing culture compared to methane growth was revealed by metabolomics analysis. In methanol-growing cells, the TCA cycle is incomplete and *de novo* production of OAA and malate occurs through carboxylation reactions from pyruvate and PEP [60]. During the growth on methanol, the main function of the TCA cycle is to provide precursors for *de novo* biosynthesis and the amount of NADH generated by this pathway decreases.

The stimulation of PEP formation from malate in the cell-free extract by high NAD^+^ concentration suggests that MaDH and PPi-dependent PEPCK can participate in malate catabolism in *Mtm*. *alcaliphilum* 20Z and corroborates the reversibility of the PEPCK reaction. As a malic enzyme, PEPCK may be responsible for the withdrawal of C4-intermediates from the TCA cycle and thus fulfil the catabolic function in cataplerosis. However, high NAD^+^ concentration was required for the malate → OAA → PEP conversion due to the low MaDH affinity towards this co-factor. Inhibition of the Mae activity by NH_4_^+^ indicates that the availability of sufficient amount of ammonium nitrogen in the cells can prevent the malate catabolism via malic enzyme.

Further biochemical characterization of other members of the pyruvate-PEP-OAA-malate node and the transcriptomic and metabolomic analyses of *Mtm*. *alcaliphilum* 20Z would help determine the regulatory mechanisms of carbon flux through the competing pathways.

## Conclusion

The analysis of biochemical properties of the *Mtm*. *alcaliphilum* 20Z Mae gives some insight into its possible metabolic role in this organism and other closely related methanotrophs. The inability to carboxylate pyruvate and the low affinity for malate imply that the main function of Mae to remove excess malate. Likewise, the inhibition of the *Mtm*. *alcaliphilum* Mae by NH_4_^+^ indicates that nitrogen limitation/excess could regulate the carbon flux through the TCA cycle. The mode of distribution of two phylogenetically different forms of the malic enzyme not correlating with the taxonomic position of host organisms suggests the high variety of enzyme functions in the central metabolism of methanotrophs.

## Supporting information

S1 Fig The scheme of obtaining the pCM:mae-Km plasmid for insertional inactivation of the *mae* gene. (TIF)

S2 Fig Gel electrophoresis of PCR products obtained with the primers Mae-Acc-Nde-F and Mae-Xho-R using template DNA: From the strain with complementation of the mae- mutation (*mae^-^::mae*) (lane 1), from *mae^-^* mutant (lane 2), from the strain with complementation of the *mae^-^* mutation (*mae^-^::mae*) (lane 3), from the wild type strain (lane 4), plasmid pMHA:mae (lane 5) and plasmid pCM:mae-Km (lane 6). M, molecular mass markers GeneRulerTM DNA Ladder Mix (Thermo Scientific).
(TIF)

S3 Fig Non-denaturating gel electrophoresis in the pore-limiting gradient of polyacrylamide (4–30%). 1, enzyme mix (top down): Mae from *Ms*. *trichosporium* OB3b (488 kDa; Rozova et al., 2019), pyruvate kinase II from *Mtm*. *alcaliphilum* 20Z (350 kDa; Kalyuzhnaya et al., 2013), Mae from *Mtm*. *alcaliphilum* 20Z (260 kDa; this work) and malate dehydrogenase from *Mtm*. *alcaliphilum* (140 kDa; Rozova et al., 2015); 2, Mae from *Mtm*. *alcaliphilum*. M, molecular mass markers.
(TIF)

S4 Fig The effect of pH on activity of Mae from *Mtm. alcaliphilum* 20Z (in percent of maximal activity). *Circle*, MES-NaOH buffer; *square*, K-phosphate buffer; *triangle*, Tris–HCl buffer; *rhomb*, Glycine-NaOH buffer.
(TIF)

S5 Fig Influence of temperature on the *Mtm. alcaliphilum* Mae activity. (TIF)

S6 Fig Analysis of products of the reactions catalyzed by cell-free extracts of the WT strain and *mae^-^* mutant of *Mtm. alcaliphilum* 20Z. (A) Release time and absorption standards for organic substances. (B) The chromatogram of incubation mixture with fumarate as a substrate. The red line represents reaction products in cell-free extract of the WT strain; the black line represents reaction products in cell-free extract of the *mae*^-^ mutant; the green line represents reaction products in cell-free extract of the strain with complementation of the *mae*^*-*^ mutation (*mae*^*-*^::*mae*). (C) The chromatogram of incubation of WT cell-free extracts with malate (violet) or malate + NAD^+^ (red). (D) The chromatogram of incubation mixture with OAA as a substrate. Red–cell-free extract of the WT strain, black–cell-free extract of the *mae*^-^ mutant, orange–control mixture without cell-free extract containing 5 mM oxaloacetate, 0.3 mM NADH, 50 mM Tris-HCl.
(TIF)

S7 Fig Alignment of the amino acid sequences of the NAD^+^-Mae from *E. coli* (AUG16249), *Mtm. alcaliphilum* 20Z (CCE22813), Ascaris suum (ADY42648), human mitochondrial NAD^+^-Mae (AAA36197), and the NADP^+^-Mae from *E. coli* (AUG17207), *Ms. trichosporium* (2507408727) and *T. kodakaraensis* (BAE47514). The chimeric malic enzymes from *E*. *coli* and *Ms*. *trichosporium* OB3b are shown without the *patr*-fragment. The square denotes highly conserved dinucleotide binding sequences.
(TIF)

S1 Table Influence ions on the *Mtm. alcaliphilum* Mae. (TIF)
