# Supplementary figures and images for: Role of the malic enzyme in metabolism of the halotolerant methanotroph Methylotuvimicrobium alcaliphilum 20Z
Source: PLoS One. 2019 Nov 18;14(11):e0225054. doi: 10.1371/journal.pone.0225054 (PMC6860931; doi:10.1371/journal.pone.0225054)

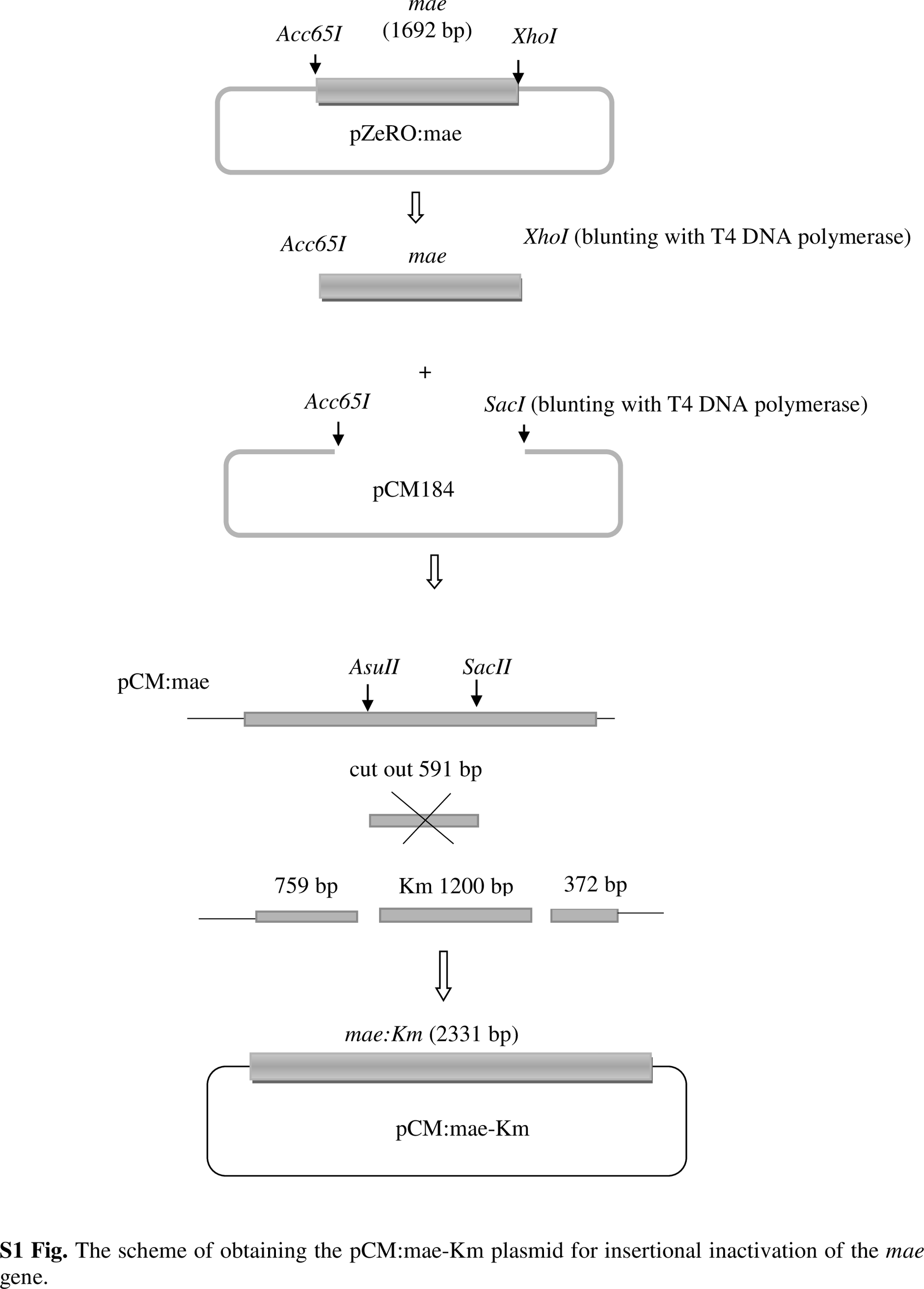

Supplement: S1 Fig — (TIF) [file pone.0225054.s001.tif]

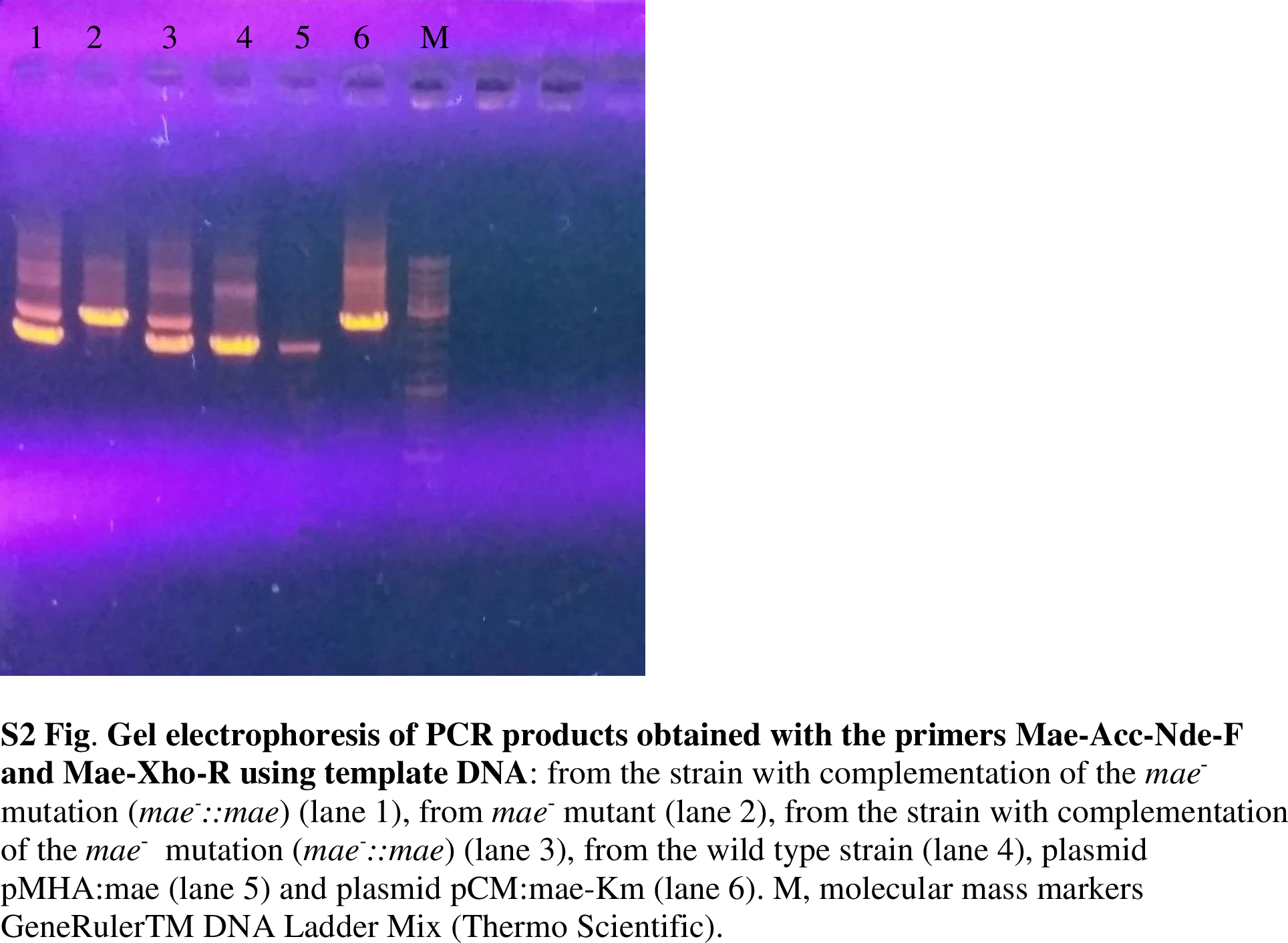

Supplement: S2 Fig — M, molecular mass markers GeneRulerTM DNA Ladder Mix (Thermo Scientific). (TIF) [file pone.0225054.s002.tif]

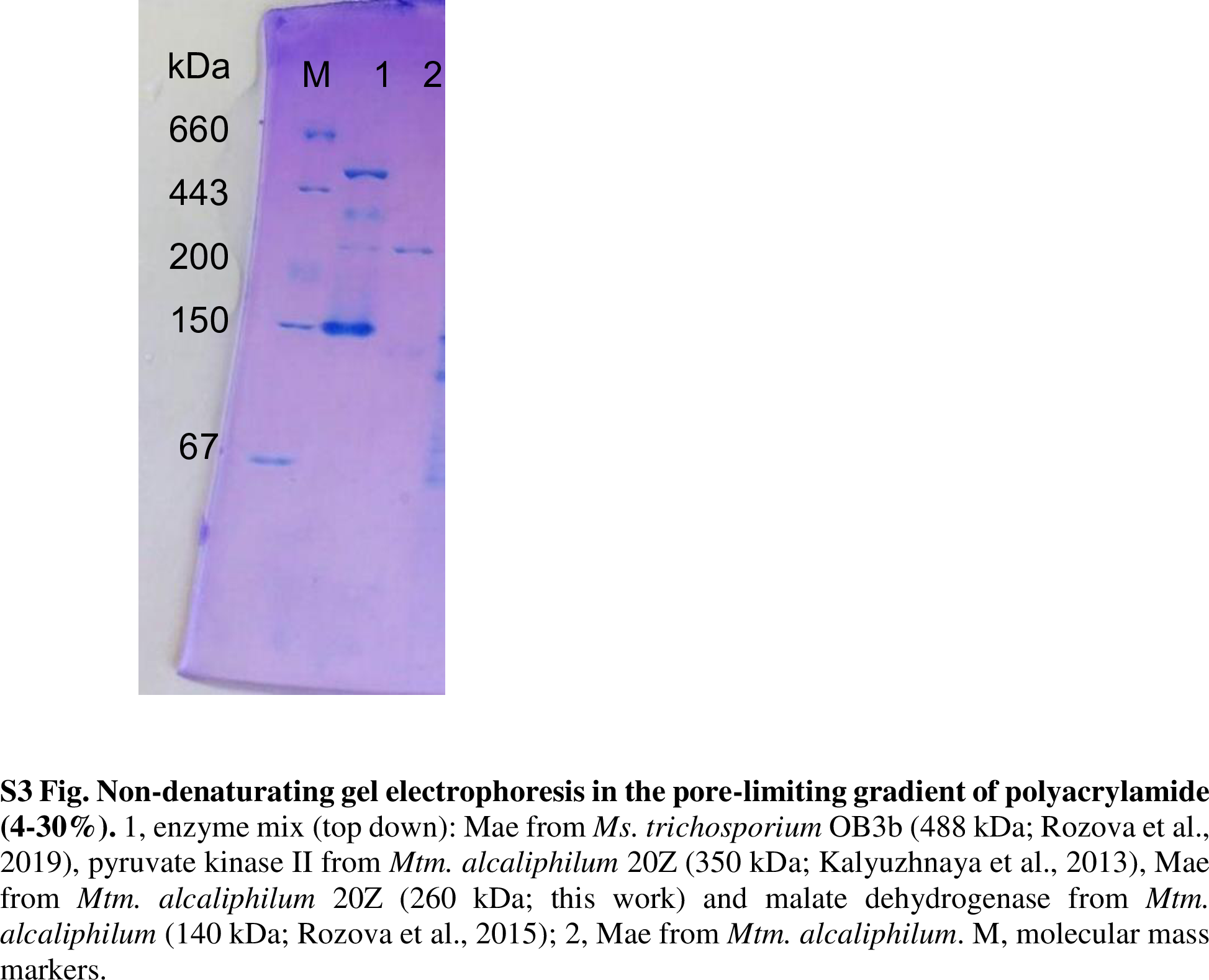

Supplement: S3 Fig — 1, enzyme mix (top down): Mae from Ms. trichosporium OB3b (488 kDa; Rozova et al., 2019), pyruvate kinase II from Mtm. alcaliphilum 20Z (350 kDa; Kalyuzhnaya et al., 2013), Mae from Mtm. alcaliphilum 20Z (260 kDa; this work) and malate dehydrogenase from Mtm. alcaliphilum (140 kDa; Rozova et al., 2015); 2, Mae from Mtm. alcaliphilum. M, molecular mass markers. (TIF) [file pone.0225054.s003.tif]

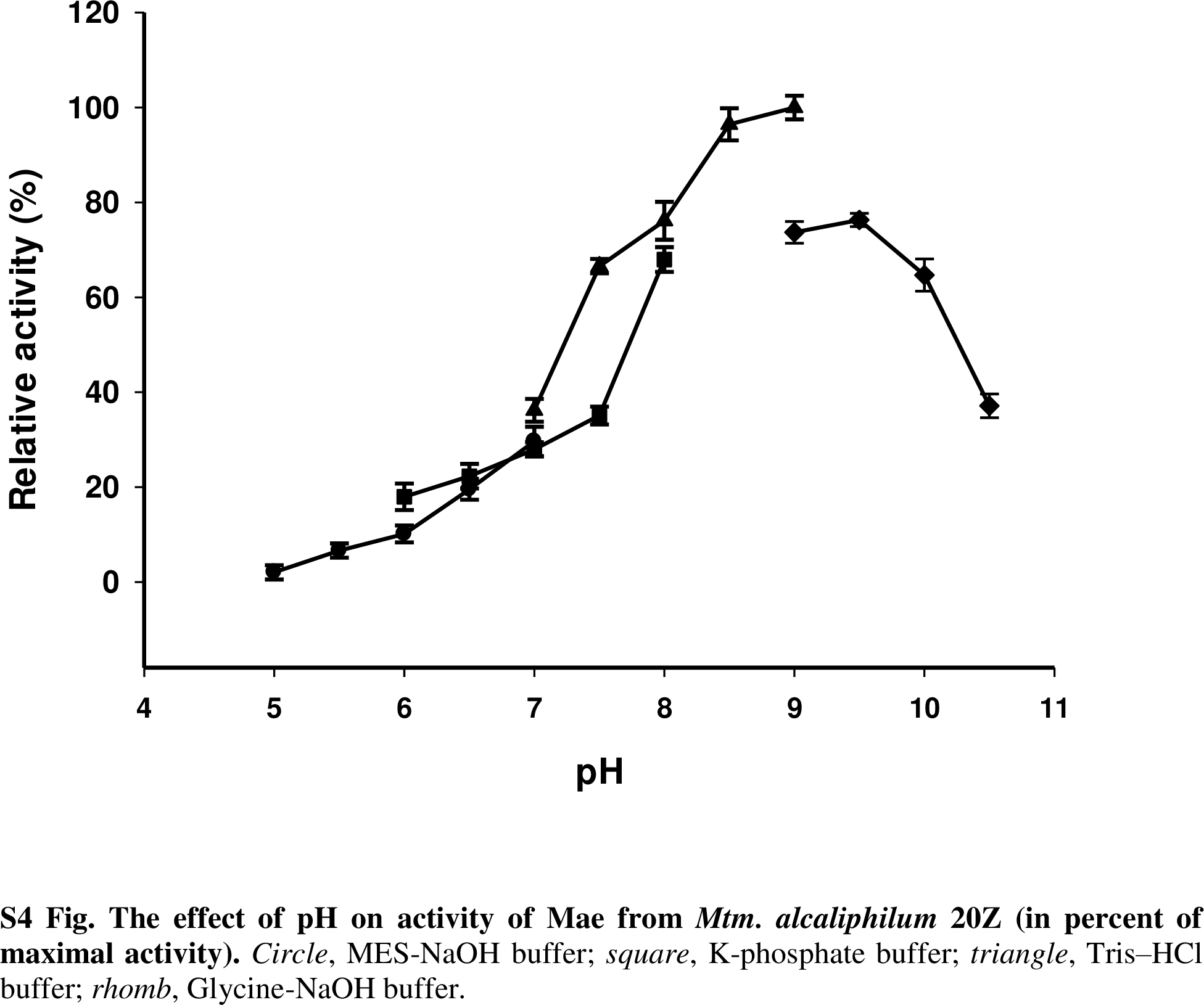

Supplement: S4 Fig — Circle, MES-NaOH buffer; square, K-phosphate buffer; triangle, Tris–HCl buffer; rhomb, Glycine-NaOH buffer. (TIF) [file pone.0225054.s004.tif]

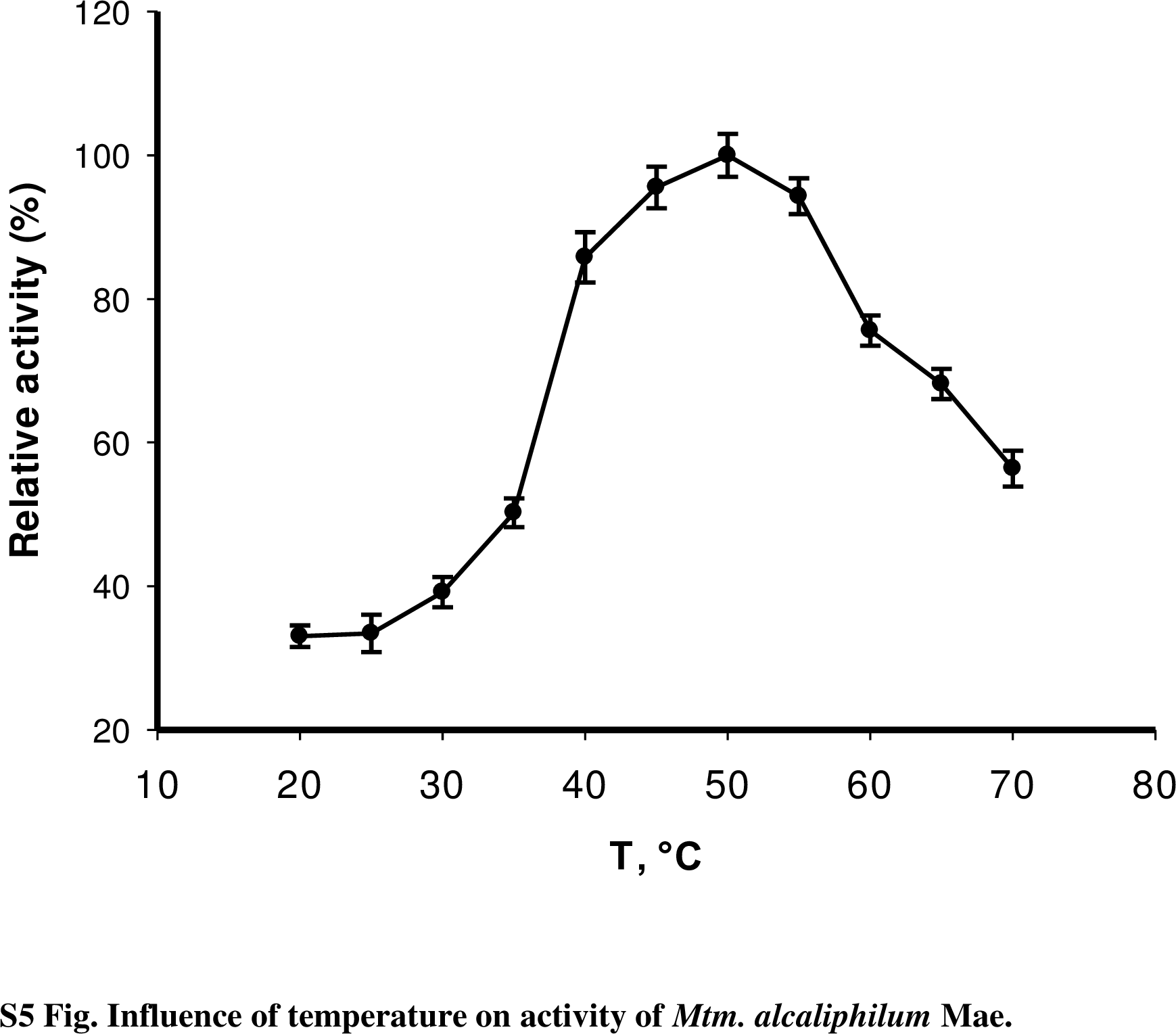

Supplement: S5 Fig — (TIF) [file pone.0225054.s005.tif]

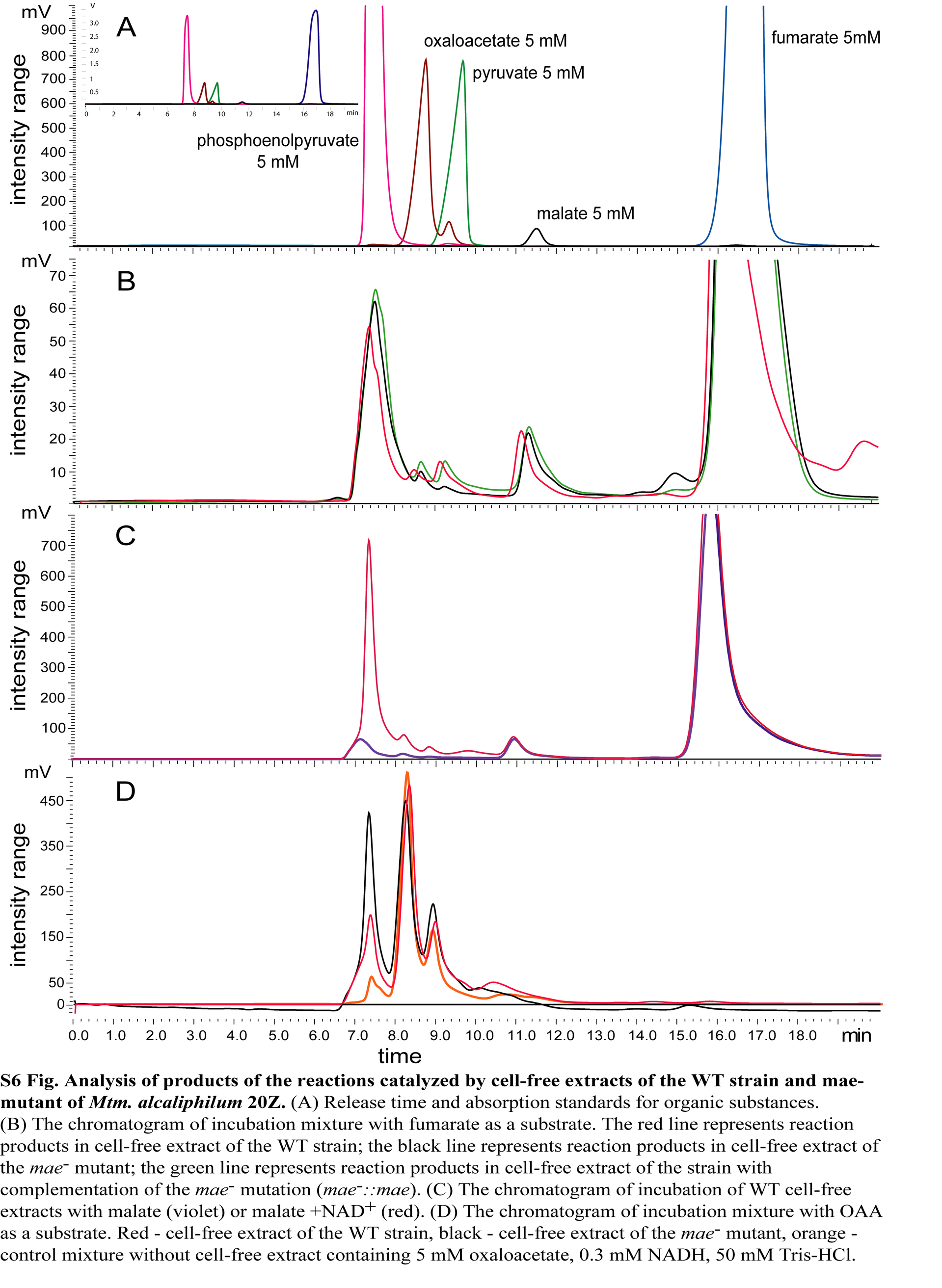

Supplement: S6 Fig — (A) Release time and absorption standards for organic substances. (B) The chromatogram of incubation mixture with fumarate as a substrate. The red line represents reaction products in cell-free extract of the WT strain; the black line represents reaction products in cell-free extract of the mae- mutant; the green line represents reaction products in cell-free extract of the strain with complementation of the mae- mutation (mae-::mae). (C) The chromatogram of incubation of WT cell-free extracts with malate (violet) or malate + NAD+ (red). (D) The chromatogram of incubation mixture with OAA as a substrate. Red–cell-free extract of the WT strain, black–cell-free extract of the mae- mutant, orange–control mixture without cell-free extract containing 5 mM oxaloacetate, 0.3 mM NADH, 50 mM Tris-HCl. (TIF) [file pone.0225054.s006.tif]

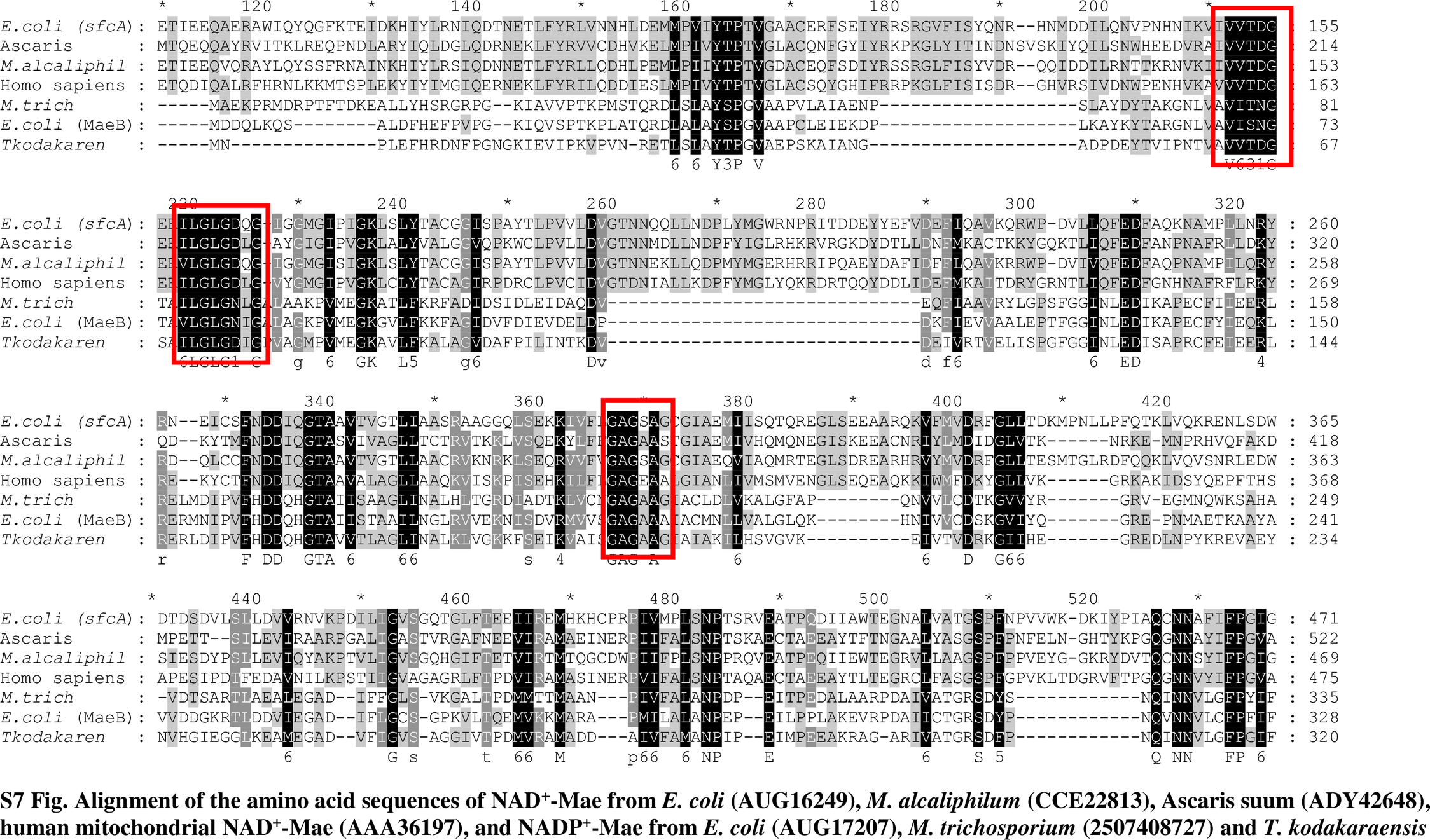

Supplement: S7 Fig — The chimeric malic enzymes from E. coli and Ms. trichosporium OB3b are shown without the patr-fragment. The square denotes highly conserved dinucleotide binding sequences. (TIF) [file pone.0225054.s007.tif]

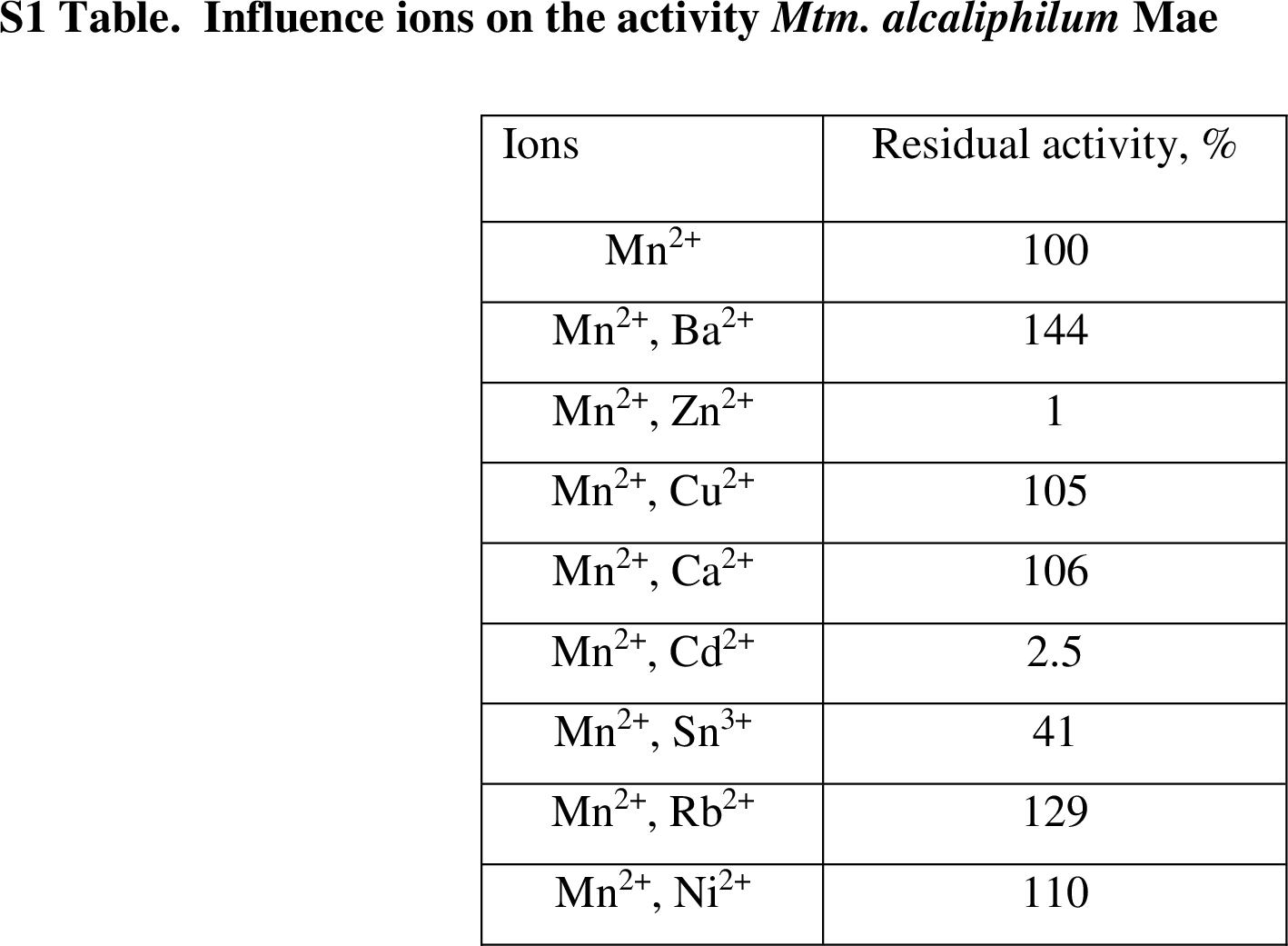

Supplement: S1 Table — (TIF) [file pone.0225054.s008.tif]
